# Enhance summer stress resilience and photosynthetic efficiency of grapevines through foliar application of silicon and kaolin combination

**DOI:** 10.1186/s12870-026-09491-w

**Published:** 2026-07-14

**Authors:** Ana I. Monteiro, Sandra Pereira, Helena Ferreira, Renata Moura, Zelia Branco, Luís Felix, Bruno Soares, Manuel João Oliveira, Igor Gonçalves, Leonor Pereira, Joana Valente, Fernando Alves, João Coutinho, José Moutinho-Pereira, Lia-Tânia Dinis

**Affiliations:** 1 CITAB - Centre for the Research and Technology of Agro-Environmental and Biological Sciences, University of Trás-os-Montes and Alto Douro (UTAD), Quinta de Prados, Vila Real, 5000-801 Portugal; 2 Inov4Agro - Institute for Innovation, Capacity Building and Sustainability of Agri-Food Production, University of Trás-os-Montes and Alto Douro (UTAD), Quinta de Prados, Vila Real, 5000-801 Portugal; 3 ADVID - Vine and Wine Cluster and CoLAB Vines &Wines, Régia Douro Park, Vila Real, 5000-033 Portugal; 4 Symington Family Estates, Travessa Barão de Forrester 86, Vila Nova de Gaia, 4400-034 Portugal

**Keywords:** Mediterranean viticulture, Summer stress tolerance, Mitigation strategies, Sustainability, Photosynthesis, Secondary metabolism, Water status, Yield

## Abstract

**Background:**

Climate change is increasingly exposing vineyards to higher temperatures and water deficits, negatively affecting grapevine physiological performance and berry composition. These effects are particularly evident in Mediterranean regions such as the Douro Demarcated Region, where summer stress often limits yield and quality.

**Materials and methods:**

In this context, this study evaluated four foliar applications combining 2% kaolin (Kl) with silicon (Si) concentrations ranging from 2% to 8% (MiKS) and their effects on physiological and biochemical responses and water status of *Vitis vinifera* cv. “Touriga Nacional”, with MiKS 1- MiKS 3 evaluated in 2023, while MiKS 2- MiKS 4 were assessed in 2024 to explore the effect of higher silicon concentrations.

**Results:**

Foliar application of MiKS formulations, especially MiKS 3 (2% Kl + 6% Si), improved grapevine water status during critical phenological stages, as indicated by higher predawn leaf water potential (Ψ_pd_). Gas exchange parameters showed a positive response, with increased stomatal conductance (*gs*), transpiration (*E*), and intrinsic water use efficiency (*A/gs*), particularly at midday. MiKS 2 (2% Kl + 4% Si) and MiKS 3 (2% Kl + 6% Si) were especially effective at midday, improving photosynthetic performance and stomatal regulation. Chlorophyll *a* fluorescence parameters indicated improved photosystem II performance, with higher *Φ*_*PSII*_ and *qP* and lower *NPQ*, especially in these treatments. Anatomical analysis revealed thicker leaf cuticles in MiKS-treated vines, particularly under higher silicon concentrations, suggesting a structural adaptation to reduce water loss. In addition, MiKS treatments were associated with increased chlorophyll and carotenoid contents. Lower levels of reactive oxygen species (ROS), including hydrogen peroxide (H_2_O_2_) levels, and lower proline content, suggest that MiKS 2 and MiKS 3 may contribute to mitigating oxidative stress and maintaining photosynthetic machinery integrity.

**Conclusions:**

These findings highlight the potential of combined KL and Si formulations, particularly MiKS 2 and MiKS 3 (4–6% Si), to alleviate summer stress in grapevines, improving physiological performance, structural protection and productivity, supporting their use as sustainable foliar strategies for Mediterranean viticulture.

## Introduction

Climate change poses an increasing threat to modern viticulture, particularly in Mediterranean regions such as Portugal. In recent years, viticulture has faced unprecedented challenges, including rising average annual temperatures, prolonged drought periods, and extreme weather events such as heat waves and heavy rainfall at unusual times [1, 2]. According to climate projections, average temperatures in Mediterranean regions are expected to rise by 2 to 5 °C (SSP5-8.5) by the end of the 21st century (2081–2100), accompanied by a 20 to 40% reduction in annual precipitation [3].

Although grapevines are known for their adaptability to adverse environmental conditions, these climatic phenomena significantly impact plant physiology, inducing substantial changes in key metabolic processes such as photosynthesis, transpiration, and respiration. As a result, growth may be constrained, leading to reduced productivity and, consequently, affecting both the oenological quality of grapes and the profile of the wines produced (e.g., elevated sugar content, lower acidity, and reduced aromatic complexity [4–7].

Given these challenges, it is imperative to implement sustainable strategies that mitigate these negative effects, enhancing vine resilience and preserving yield quality, aligning with the sustainable development goals (SDGs) of the 2030 Agenda, namely SDG 13 (Climate Action) and SDG 12 (Responsible Consumption and Production), and SDG 15 (Life on Land).

In this context, recent studies have explored the physiological and biochemical responses of grapevines to foliar applications of exogenous compounds, particularly kaolin (Kl) and silicon (Si), applied either individually, as well as their effects on grape quality and wine profile [8–10]. The studies have contributed to a better understanding of the mechanisms underlying grapevine adaptation to environmental stress and their relationship with quality wine [9–13].

Among the strategies evaluated, foliar application of Kl has emerged as an effective approach to mitigating heat stress in plants. This aluminium silicate (Al₂Si₂O₅(OH)₄), is a natural, inert, and non-toxic compound, that forms a porous, whitish particle film on the leaf surface. This film allows gas exchange while efficiently reflecting infrared and ultraviolet radiation [14–16].

By reducing heat load, Kl minimizes the risk of sunburn damage to leaves and fruits under high irradiance conditions [15–17]. The resulting decrease in leaf temperature, together with improved water retention, contributes to the maintenance of photosynthetic and stomatal activity under water and heat stress conditions, thereby enhancing plant resilience and preserving productivity [18–20].

Although numerous studies have demonstrated the effectiveness of Kl in improving grapevine resilience under harsh environmental conditions in various crops, including apple [21], olive [22, 23], and citrus [24], there is growing interest in enhancing its efficacy through combination with other compounds, such as Silicon (Si) Indeed, Si application has demonstrated notable benefits in mitigating abiotic and biotic stress in several crops, including tomato [25], beans [26], and grapevines [9, 27].

This element act through complementary mechanism. On one hand, it may contribute to the reinforcement of cell walls following foliar absorption, strengthening cellular structure and potentially reducing stomatal conductance and transpiration, thereby limiting water loss under drought and heat stress. On other hand, Si regulates biochemical responses, including oxidative stress pathways, by enhanced antioxidant enzymes activity, and regulating photosynthetic processes such as Rubisco activity. Together, these effects may help protect against oxidative damage while maintaining photosynthetic efficiency [28–30]. Building on this knowledge, the present study aims to provide insights to support the development of sustainable viticultural strategies to mitigate prolonged summer stress, with the potential to enhance grapevine resilience and maintain quality and productivity under increasingly adverse climatic conditions.

This study aims to investigate the combined effect of Kl-Si formulations (MiKS 1- MiKS 4) on improving grapevine tolerance to prolonged summer stress, particularly heat and drought stress, which are characteristic of Mediterranean climate. Key physiological and biochemical parameters were assessed, including gas exchange, leaf biochemical composition, and plant water relations.

### Experimental site and plant material

The present study was conducted in a commercial vineyard located at “Quinta do Bomfim” (41º11′21.28″N, 7º32′27.40″W) in Pinhão, Cima Corgo sub-region of Douro Demarcated Region, Portugal, over two growing seasons between June and September of 2023 and 2024.

The experimental vineyard was planted in 2015 and consists of cultivated grapevines (*Vitis vinifera* L.) cultivar “Touriga Nacional”, grafted onto the R110 rootstock, and managed under standard commercial viticultural practices. Leaves were collected from cultivated plants; therefore, no specific permissions or licences were required for sample collection. No voucher specimens were deposited, as the study involved a grapevine cultivar widely cultivated and representative of the region, grown under local legislation commercial vineyard conditions in accordance with the applicable legislation and regulations. Row and vine spacing were 2.00 m and 1.00 m, respectively, and plants were trained using a unilateral Royat cordon with vertical shoot positioning (VSP) in an east–southeast to west-northwest direction, with 3 to 4 spurs (6 to 8 pruning load/grapevine). According to the Köppen-Geiger classification, the area is classified as Csb, a temperate climate with dry and hot summers, as well as cold conditions with moderate precipitation during the winter months [31].

**Fig. 1:**
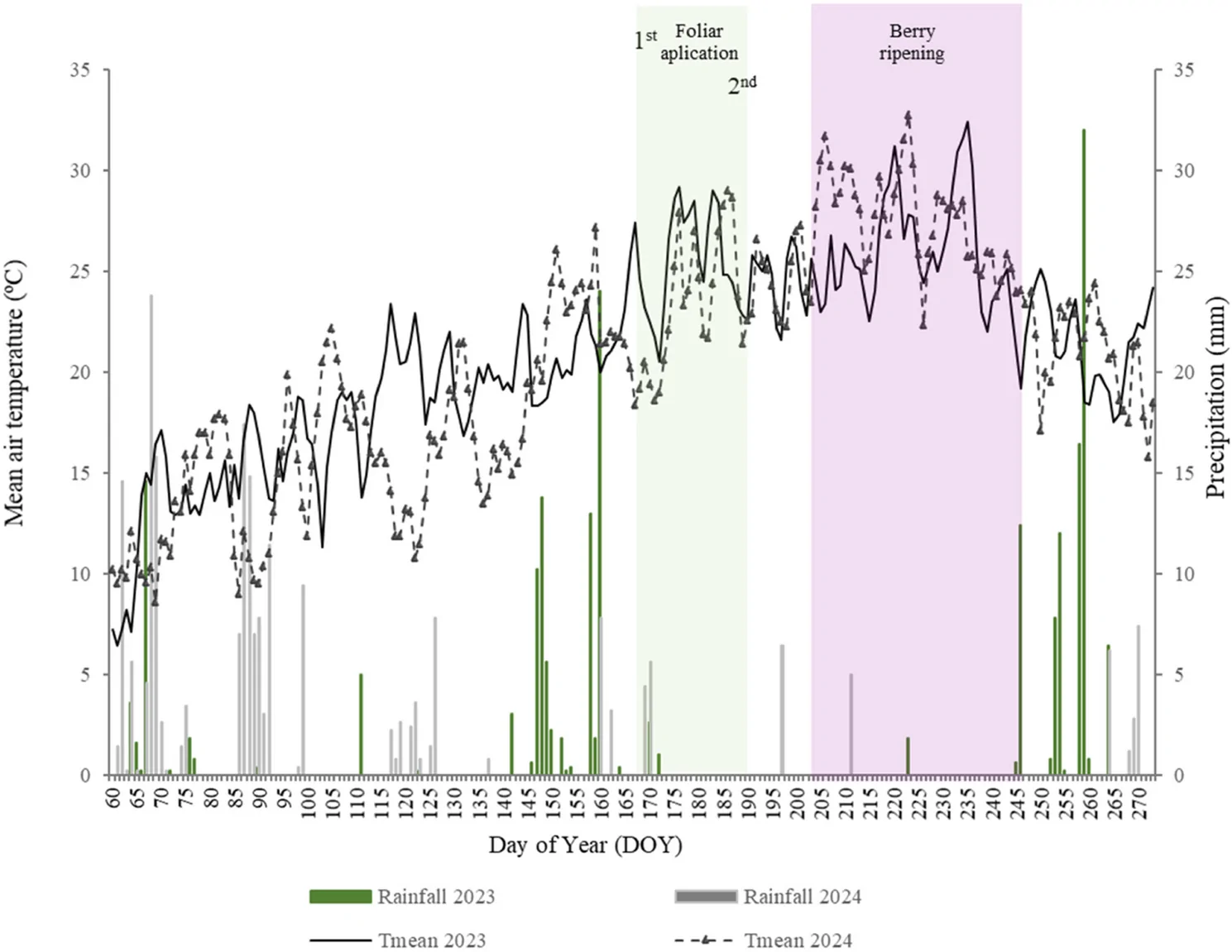
Daily mean air temperature (Tmean, represented by the lines), and precipitation (bars) recorded in Quinta do Bomfim (Pinhão) from 1^st^ April (DOY 91) to 30th September (DOY 273) for the years 2023 and 2024. DOY (Day of the Year)

The meteorological data were automatically collected from an automatic weather station near the experimental plot. Data from two growing seasons, including precipitation and maximum and average temperatures by day of the year (DOY), is presented in Fig. 1. The average air temperature from April 1 (DOY 91) to September 30 (DOY 273) indicated an average daily air temperature of 22.3 °C in 2023, which decreased slightly to 21.9 °C in 2024. The number of days with temperatures exceeding 35 °C increased significantly, from 25 in 2023 to 35 days in 2024. Additionally, the occurrence of days with temperatures above 40 °C showed a slight increase, from 4 in 2023 to 6 days in 2024.

During the same period, accumulated precipitation decreased from 177.0 mm in 2023 to 93.6 mm in 2024, representing 30.3% and 18.3% of the annual total, respectively. The Dryness index (DI) for this region was 22.2 in 2023 and − 43.4 in 2024. According to the classification of [32] both values fall within the “moderately dry” category (DI + 1), indicating that grapevines in the region experienced moderate water deficit, with greater severity in 2024. Similarly, the Huglin index used to evaluate heat availability during the growing season increased from 2976.2 (2023) to 3986.5 (2024), corresponding to a classification of warm climate (HI + 2) to very warm climate (HI + 3), which translates into more challenging conditions for grapevine development [32, 33].

### Vineyard foliar treatments application

The experimental design consisted of three randomized blocks, each comprising four subplots (rows), corresponding to the four analysed treatments, with 20 grapevines per subplot, totalling 240 plants. Each subplot (row), correspond to one treatment and was considered the experimental unit. Each treatment was replicated across the three blocks, resulting in three independent replicates (*n* = 3).

**Fig. 2:**
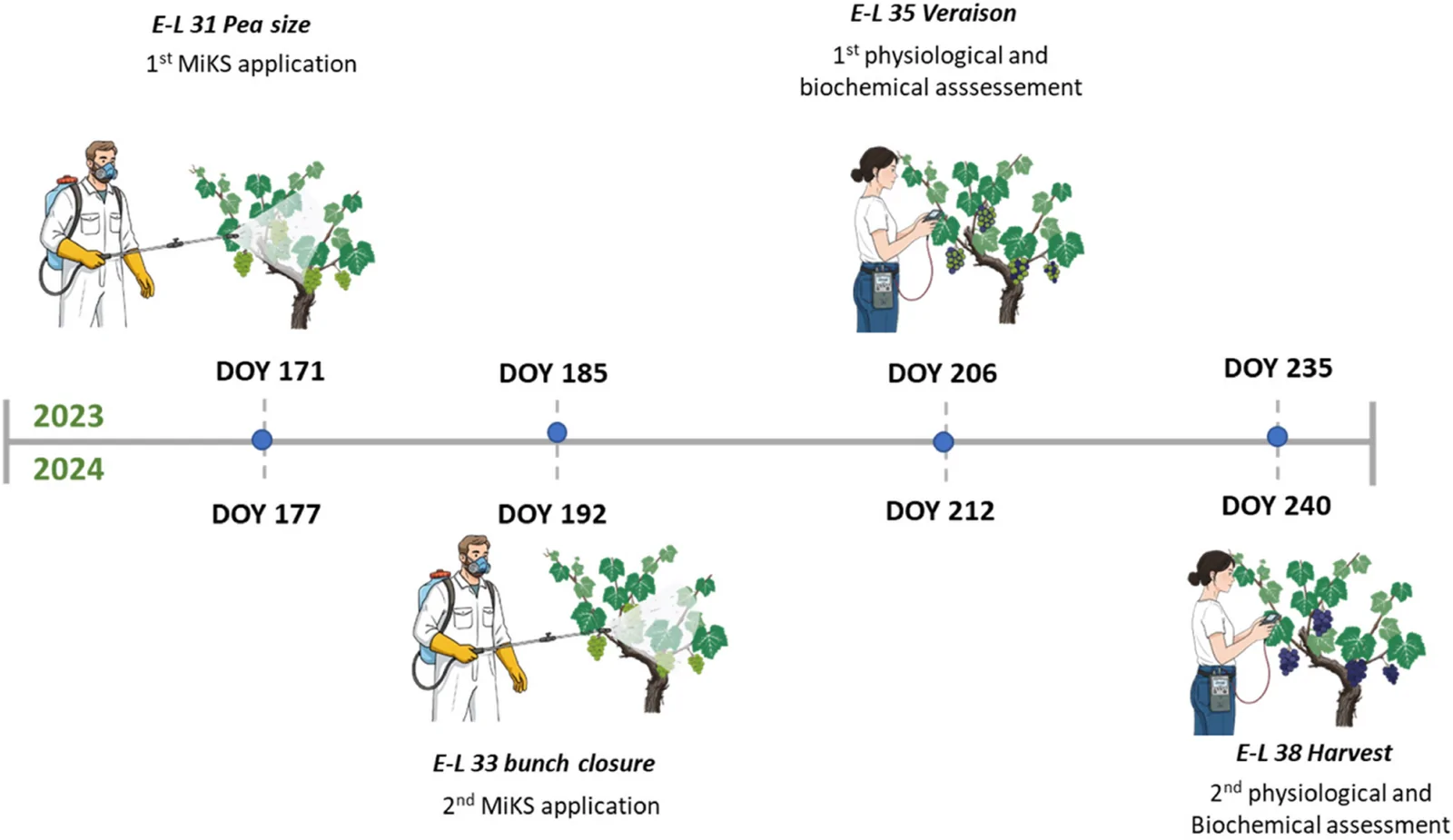
Timeline of foliar applications and ecophysiological measurements during the two growing seasons (2023 and 2024)

In the first year, four experimental treatments were applied: (i) Control (CTRL, only water application), and three formulations combining 2% kaolin (Kl) with silicon (Si) at concentrations of 2%, 4% and 6%, corresponding to (ii) MiKS 1 (2% kaolin + 2% silicon); (iii) MiKS 2 (2% kaolin + 4% silicon), and (iv) MiKS 3 (2% kaolin + 6% silicon), based on proportions previously reported by [9]. In the second year (2024), the experimental design was adjusted to further explore the effects of higher silicon concentrations. The MiKS1 treatment was discontinued and replaced by MiKS4, a formulation containing 2% kaolin (Kl) and 8% silicon (Si), while the remaining treatments (CTRL, MiKS2, and MiKS3) were maintained. The experimental formulations were prepared using commercial products, Humigel Kaolin and HumigelPlus A (Tecniferti, Portugal). The silicon source (HumigelPlus A) is a liquid formulation supplying soluble silicon, expressed as silicon dioxide (SiO₂, 6% w/w, according to the manufacturer’s specification). Spray solutions were prepared by first diluting the silicon formulation in water, then gradually addicting Humigel Kaolin under continuous agitation to obtain a homogeneous suspension. No additional adjuvants or surfactants were included. For each treatment, 15 L of spray solution was prepared and applied using a manual sprayer, corresponding to approximately 0.25 L per plant (60 plants per treatment). Spraying was performed uniformly across treatments until near runoff, ensuring c uniform canopy coverage and visible deposition of the kaolin-based film on the leaf surface. Two foliar applications were performed per season: the first at pea-size (E-L 31), followed by a second application 15 days later, as shown in Fig. 2. All physiological measurements and leaf samples for biochemical analyses were performed on healthy leaves from the middle third of the canopy, fully expanded, and sun-exposed on DOY 206 (E-L 35, Veraison) and DOY 235 (E-L 38, Harvest), in 2023 and DOY 198 (E-L 35, Veraison) and DOY 240 (E-L 38, Harvest) in 2024 (Fig. 2).

### Physiological parameters

#### Leaf water status

Predawn leaf water potential (Ψ_pd_) was measured before sunrise in fully expanded and healthy leaves, using a pressure chamber (PMS Instrument Company, Model 1000, Albany, USA) and following the method described by [34]. Measurements were performed on six plants per treatment (*n* = 6), corresponding to two plants per subplot, using one leaf per plant.

#### Gas exchange and chlorophyll *a* fluorescence

Leaf gas exchange was measured using an infrared gas analyzer (IRGA, LCPro+, ADC Bioscientific Ltd., Hoddesdon, UK) operating in open mode. Measurements were performed on six plants per treatment (*n* = 6), corresponding to two plants per subplot, using one fully expanded, sun-exposed leaf per plant.

Data were collected during two time periods: morning (9:00–10:30 a.m.) and midday (2:00–3:30 p.m.). The following parameters were calculated: stomatal conductance (*g*_*s*_), and transpiration rate (*E*), based on the methodology of von Caemmerer and Farquhar (1981). Chlorophyll *a* fluorescence measurements were carried out at the same time and on the same leaves used for IRGA at each developmental stage using a Pulse Amplitude Modulation Fluorometer (mini-PAM, Photosynthesis Yield Analyzer; Walz, Effeltrich, Germany). The maximum quantum efficiency of photosystem II (PSII) was calculated as *F*_*v*_*/F*_*m*_ = (*F*_*m*_-*F*_*0*_)/*F*_*m*_ by measuring fluorescence in dark-adapted leaves when all reaction centers were open (*F*_*0*_) and during a saturating light pulse (0.7 s at 15.000 µmol·m⁻²·s⁻¹ white light), when all reaction centers were closed (*F*_*m*_). Leaves were dark-adapted for 30 min using specific leaf clips (by a Dark Leaf Clip DLC-8) before measurements.

After determining *F*_*v*_/*F*_*m*_, leaves were exposed for 20 s to actinic light (1.500 µmol·m⁻²·s⁻¹) to measure steady-state fluorescence under light-adapted conditions (*F*_*s*_), averaged over 2.5 s. A subsequent saturating light pulse (15.000 µmol·m⁻²·s⁻¹, 0.7 s) was applied to determine F′m, followed by shading for 5 s with far-red light to measure *F*_*0*_′.

Several fluorescence parameters were calculated [35, 36]: photochemical quenching (*qP* = (*F’*_*m*_– *F*_*s*_)/(*F’*_*m*_–*F’*_*0*_)),non-photochemical quenching (*NPQ* = (*F*_*m*_ - *F’*_*m*_)/*F’*_*m*_) and photosynthetic electron transport rate *ETR* (µmol electrons m^‒2^ s^‒1^) = (*ΔF*/*F’*_*m*_) × PPFD × 0.5 × 0.84, where PPFD is the photosynthetic photon flux density incident on the leaf, 0.5 is the factor that assumes equal distribution of energy between the two photosystems, and the leaf absorbance used was 0.84 a commonly used value for C_3_ plants.

### Leaf structural by microscopy and digital image analysis

Histological analysis was performed on six healthy fully expanded leaves per treatment (*n* = 6), corresponding to two plants per subplot, each collected from independent plants. Leaf samples were collected from the central region to minimize thickness variability. Transverse Sect.  (4 μm) were obtained using a rotary microtome (Leica RM 2135, Germany) after fixation in FAA (24 h), dehydration, clearing, and paraffin embedding. Sections were mounted on slides and stained with 0.1% toluidine blue. Tissue thickness was quantified in transverse sections using an inverted optical microscope (Olympus IX51, Olympus Corporation, Tokyo, Japan) and Digimizer image analysis software (MedCalc Software’s, Ostend, Belgium).

### Leaf biochemical analysis

Photosynthetic pigments and metabolic assays were analyzed according to [10] using six mature (*n* = 6, two plants per subplot), fully expanded leaves per foliar treatment, collected at the ripening stage between 10:00 and 11:00 a.m. Leaves were immediately frozen in liquid nitrogen and stored at − 80 °C until analysis.

The quantification of chlorophylls (chlorophyll *a* (Chl *a*), chlorophyll *b* (Chl *b*), total chlorophyll (Chl_(*a+b*)_) and carotenoid (Car) were extracted following the methodology described by [37]. Both determinations were performed using 80% cold acetone with distilled water (*v*/*v*) and under dim light to avoid photosynthetic pigment degradation.

The leaf phenol compounds assay was performed in triplicate, extracted in MeOH: H_2_O (70:30, v/v) at an initial concentration of 4 mg ml^− 1^, Total phenols were determined according to the Folin-Ciocalteu’s procedure with absorbance measured at 725 nm, and expressed as mg GAE g⁻¹ DW [38]. Total soluble proteins were quantified using the method of [39] using bovine serum albumin as a standard, and expressed as mg g^− 1^ FW. Soluble sugars were determined by the anthrone-sulfuric acid method adapted from [40], and results were expressed as mg g^− 1^ FW. The proline content was extracted with 3% (w/v) sulfosalicylic acid (SSA) and centrifuged at 12,000 g for 15 min at 4 °C, as described by [41].

Reactive Oxygen Species (ROS) were quantified using 2′,7′-dichlorofluorescein diacetate (DCFH-DA) (Sigma–Aldrich, Germany), following the methodology of [42].

Hydrogen peroxide (H_2_O_2_) concentration was measured following the method of [43], using a standard curve of H_2_O_2,_ and the values were expressed as µmol g^− 1^ FW.

### Leaf mineral composition

Leaf petioles (*n* = 15 per treatment) were collected from independent plants at veraison, with five petioles obtained from each subplot. Nutrient concentration was determined in dried 70 °C, and ground material. Nitrogen was analysed in an elemental analyser (Primac CN 100, Skalar Analytical) by combustion at 1000 °C and quantified by a thermal conductivity detector (ISO 16634). The remaining nutrients were determined after an acid digestion (HNO_3_ + H_2_O_2_) assisted by microwaves (EN 13805) and quantified by ICP-OES (iCAP series 7000, Thermoscientific).

### Yield and vine vigour

At harvest, 15 plants per treatment (5 plant per subplot) were sampled. Yield (kg per vine) was determined as total grape mass per vine by weighing all clusters per plant. The number of bunches per vine was recorded, and average bunch weight was calculated based on the weight of individual clusters. During winter, the same grapevine plants were manually pruned, and pruning weight (kg per vine) was recorded as an indicator of vegetative growth.

### Analysis statistics

Statistical analysis was performed using SPSS 29.0 software for Windows. Differences between foliar treatments were evaluated by one-way ANOVA, followed by Tukey’s post hoc test. Statistical significance was considered at *p* < 0.05. Values are expressed as mean ± standard deviation (SD). Analyses were conducted separately for each combination of season, phenological stage, and time of day, as environmental and physiological conditions differed markedly between measurement periods (morning and midday) and developmental stages (veraison and ripening).

## Results

### Changes on vines water status and physiological behaviour under foliar kaolin and silicon-based applications

**Fig. 3:**
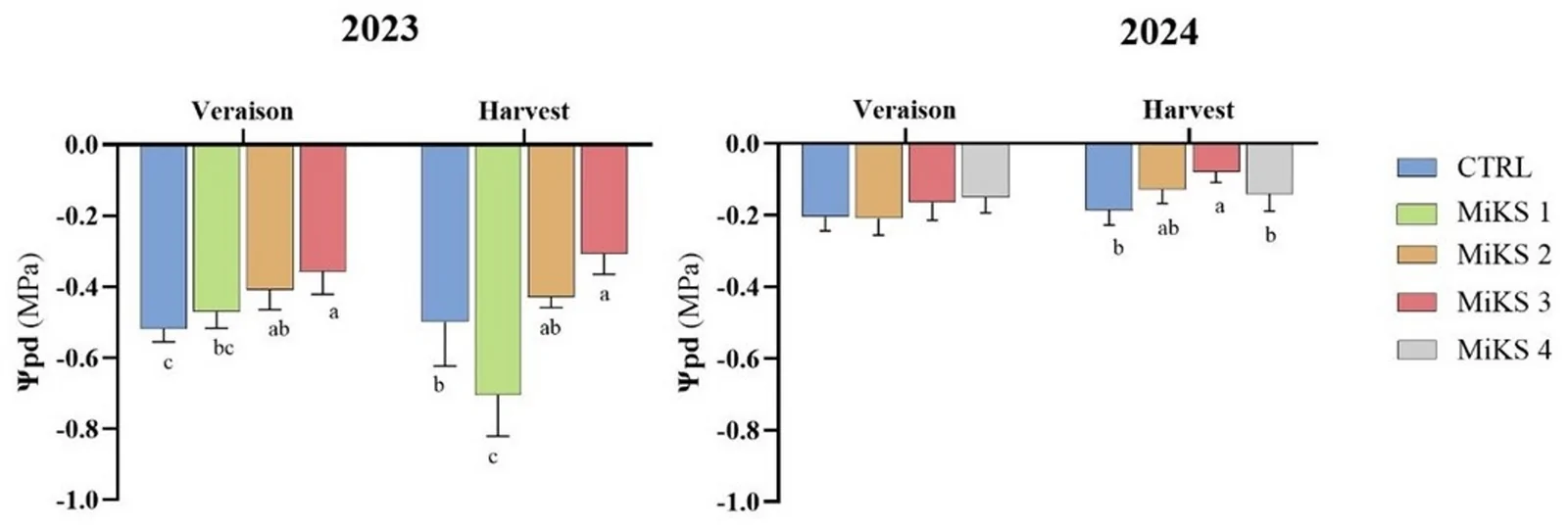
Effects of foliar application of different kaolin (Kl) and silicon (Si) formulations (MiKS): control (CTRL, water application), MiKS 1 (Kl 2%+Si 2%); MiKS 2 (Kl 2%+Si 4%); MiKS 3 (Kl 2%+Si 6%) and MiKS 4 (Kl 2%+Si 8%) on predawn leaf water potential (Ψ_pd_, MPa) of “Touriga Nacional” grapevine variety. Measurements were taken at predawn at two phenological stages (veraison and harvest) during two growing seasons: 2023 (**a**) and 2024 (**b**). Bars represent mean ± standard deviation of six replicates (*n* = 6). Different letters indicate significant differences among treatments within the same phenological stage (Tukey’s test, *p* < 0.05). The absence of letters indicates no significant differences

Predawn leaf water potential (Ψ_pd_) measurements in “Touriga Nacional” vines treated with different Kl and Si formulations (MiKS), over two growing seasons, demonstrated an overall improvement in plant water status, particularly at the ripening stage (Fig. 3). In 2023, control plants exhibited Ψ_pd_ values of -0.51 MPa at veraison, indicative of mild water deficit, due to high summer temperatures and limited precipitation [44]. All MiKS treatments showed higher Ψ_pd_ values at this stage, with MiKS 3 showing the highest value (-0.36 MPa), corresponding to an increase of 29% in Ψ_pd_ compared to control.

As summer stress intensified towards harvest, treatments effects became more evident, with MiKS 3 improving Ψ_pd_ by 36%, followed by MiKS 2 (23%), and MiKS 1 (8.1%), compared to the control vines. In 2023, Ψpd values were generally higher than in previous year. No significant differences among treatments were detected at veraison. However, at harvest, MiKS 3 treated plants exhibited the highest improvement in water status, with increases of 57% at harvest compared to the control, while MiKS 2 showed a 31% increase.

**Fig. 4:**
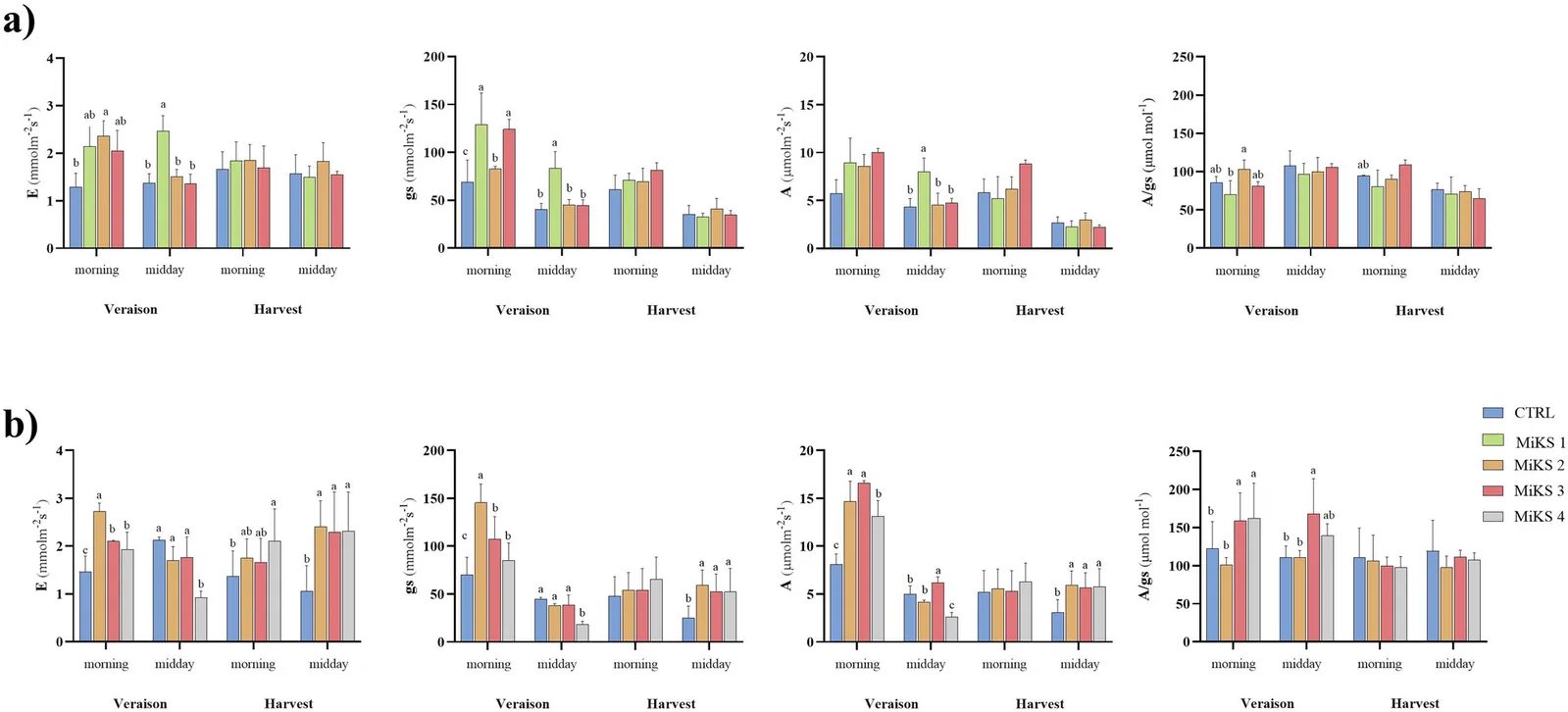
Effects of foliar application of different kaolin (Kl) and silicon (Si) formulations (MiKS): control (CTRL, water application), MiKS 1 (Kl 2%+Si 2%); MiKS 2 (Kl 2%+Si 4%); MiKS 3 (Kl 2%+Si 6%) and MiKS 4 (Kl 2%+Si 8%) on gas exchange parameters: transpiration rate (E, mmol m^− 2^ s^− 1^), stomatal conductance (g_s_, mmol m^−2^s^− 1^), net photosynthesis rate (A, µmolm^−2^s^− 1^), intrinsic water use efficiency (A/g_s_, µmol.mol^− 1^) in leaves of “Touriga Nacional” grapevine variety. Measurements were taken in two times of the day (morning − 9:00–10:30 a.m.; midday − 2:00–3:30 p.m.) at two phenological stages (veraison and harvest) during two growing seasons: 2023 (**a**) and 2024 (**b**). Bars represent mean ± standard deviation of six replicates (*n* = 6). Different letters indicate significant differences among treatments within the same phenological stage (Tukey’s test, *p* < 0.05). The absence of letters indicates no significant differences

Over two study years, gas exchange parameters were significantly influenced by foliar treatments, particularly at the veraison stage (Fig. 4). In 2023, at veraison (morning), grapevines treated with MiKS 2 showed higher *E*, *g*_*s*_ and *A/g*_*s*_ than control vines, with relative increases of 83%, 20%) and 21%), respectively.

At midday, when stress was more intense, MiKS 1 showed better performance, with higher E (2.47 to. 1.37 mmol m⁻² s⁻¹), *gs* (83.6 to 40.4 mmol m⁻² s⁻¹), and *A* (8.02 to 4.31 µmol m⁻² s⁻¹) compared to control vines, corresponding to increases of 80%, 107%, and 86%, respectively. However, no significant differences among treatments were observed in the *A/g*_*s*_. In 2024, MiKS treatments showed positive effects on gras exchange parameters.

At veraison, in the morning, vines treated with MiKS 2 shower higher *E*, *g*_*s*_ and *A*, corresponding to increase of 87%, 108% and 81%, respectively compared to the control vines. Meanwhile, MiKS 3 and MiKS 4 increased *A/g*_*s*_, up to 33%, suggesting improved physiological efficiency.

At harvest in 2024, treatments effects were more significant at midday, with MiKS 2 showing increases in *E* (from 1.06 to 2.41 mmol m⁻² s⁻¹), gs (from 24.9 to 59.5 mmol m⁻² s⁻¹), and A (from 3.06 to 5.94 µmol m⁻² s⁻¹), with relative increases of 127%, 138.8%, and 95%, respectively, compared to the control plants.

**Fig. 5:**
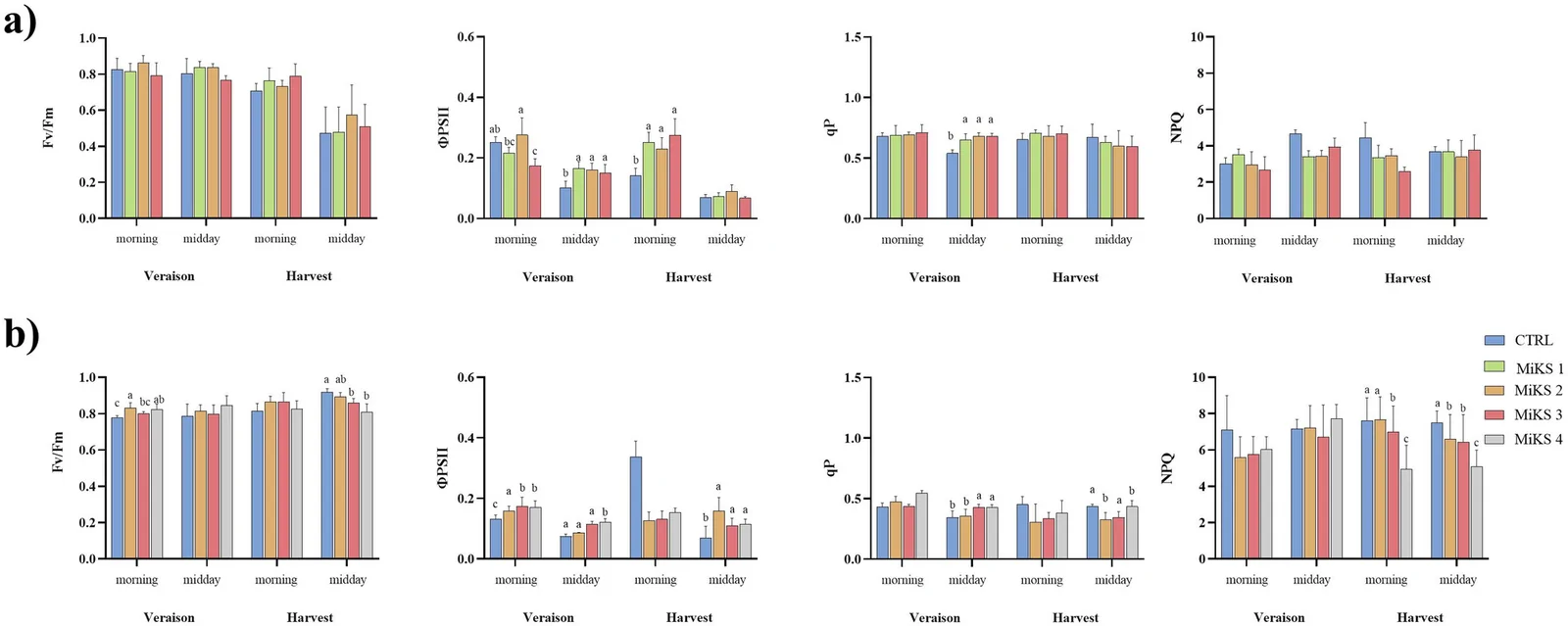
Effects of foliar application of different kaolin (Kl) and silicon (Si) formulations: control (CTRL, water application), MiKS 1 (Kl 2%+Si 2%); MiKS 2 (Kl 2%+Si 4%); MiKS 3 (Kl 2%+Si 6%) and MiKS 4 (Kl 2%+Si 8%) on chlorophyll a fluorescence parameters: maximum quantum efficiency of photosystem II (F_v_/F_m_), effective quantum efficiency of photosystem II (Φ_PSII_), photochemical quenching (qP) and nonphotochemical quenching (NPQ) of “Touriga Nacional” grapevine variety. Measurements were taken in two of the day (morning and midday) at two phenological stages (veraison and harvest) during two growing seasons: 2023 (**a**) and 2024 (**b**). Bars represent mean ± standard deviation of six replicates (*n* = 6). Different letters indicate significant differences among treatments within the same phenological stage (Tukey’s test, *p* < 0.05). The absence of letters indicates no significant differences

Regarding in vivo chlorophyll *a* fluorescence analysis, MiKS treatments improved the functioning of photosystem II (PSII). MiKS-treated plants showed better photosystem II efficiency (*Φ*_*PSII*_), a higher proportion of open reaction centres (*qP***)**, and reduced non-photochemical quenching (*NPQ*), particularly during berry ripening (Fig. 5). Overall, MiKS treated vines, particularly with MiKS 2 and MiKS 3, showed consistent response across both years. At veraison in 2023, particularly at midday, MiKS 1, MiKS 2, and MiKS 3 significantly increased *Φ*_*PSII*_ by 63.3%, 59.4% and 49.5%, respectively, compared to control vines. Similarly, these treatments increased *qP*, with up to 26% in MiKS 2 and MiKS 3, accompanied by a reduction in *NPQ*, (22.3% − 41.7%) in same treatments.

A similar pattern was observed at harvest (morning), with MiKS 3 again showing the strongest response, with increases in Φ_*PSII*_ (95%) and continued reduction in *NPQ* (42%).

In 2024, MiKS treatments also showed positive effects, highlighting their influence on PSII.

Overall, treated vines showed higher f *F*_*v*_/*F*_*m*_ (up to 6.94% for MiKS 2) and *Φ*_*PSII*_ (up to 32.8% for MiKS 3), as well as increased *qP* (up to 24% with MiKS 3 and MiKS 4) associated with a reduction in *NPQ*, particularly at midday.

At harvest, despite overall decreases, MiKS 2 and MiKS 3 treatments continued to show beneficial effects.

At midday, *Φ*_*PSII*_ was higher in MiKS plants (0.158 in MiKS 2) than in control vines (0.069). Furthermore, *qP* showed smaller variations between treatments (0.325–0.435), while NPQ was lower in treated vines, particularly in MiKS 4 (5.15) compared to control plants (7.51).

### Leaf anatomy and structural adaptation, and biochemical responses induced by kaolin and silicon mixtures formulations

Morphoanatomical parameters of “Touriga Nacional” cv. are presented in Table 1. MiKS treatments significantly modified the leaf anatomical characteristics across both growing seasons, with cuticle thickness being the most affected trait, particularly in the Si-enriched treatments (4% and 6%). This effect is particularly evident in 2023, when foliar applications led to a significant increase in upper cuticle thickness at veraison, with MiKS 2 and MiKS 3 showing increases of 52.2% and 52.7% respectively compared to control. At harvest, this pattern was observed only in MiKS 1 (18.8%), while MiKS 2 and MiKS 3 remained comparable to the control.

**Table 1:** Effects of foliar application of different kaolin (Kl) and silicon (Si) formulations (MiKS): control (CTRL, water application), MiKS 1 (Kl 2%+Si 2%); MiKS 2 (Kl 2%+Si 4%); MiKS 3 (Kl 2%+Si 6%) and MiKS 4 (Kl 2%+Si 8%) on leaf tissue thickness (µm) in “Touriga Nacional” grapevine variety at two phenological stages (veraison and harvest) during two growing seasons: 2023 and 2024. Values are expressed as mean ± standard deviation of six replicates (*n* = 6). Different letters indicate significant differences among treatments within the same phenological stage (Tukey’s test, *p* < 0.05). The absence of letters indicates no significant differences. UC: upper cuticle; UE: upper epidermis; LE: lower epidermis; LC: lower cuticle; TL: total lamina and PP/SP: palisade parenchyma/ spongy parenchyma ratio

Season	Phenological	Parameters	Foliar Treatment				*p*-value
	stage		Control	MiKS 1	MiKS 2	MiKS 3	
*2023*	Veraison	UC	2.24 ± 0.399 b	2.90 ± 0.428 a	3.41 ± 0.327 a	3.42 ± 0.246 a	< 0.001
		UE	14.2 ± 1.26 a	11.2 ± 1.57 b	12.1 ± 2.15 ab	12.9 ± 0.663 ab	0.021
		LE	11.1 ± 0.872	13.3 ± 2.54	11.7 ± 3.22	14.6 ± 2.81	0.097
		LC	2.99 ± 0.811	3.38 ± 0.269	2.83 ± 0.561	2.68 ± 0.109	0.142
		TL	128.7 ± 4.72 ab	126.8 ± 6.90 b	134.8 ± 15.2 ab	146.4 ± 13.9 a	0.027
		PP/SP	1.18 ± 0.115	1.01 ± 0.232	1.07 ± 0.084	1.12 ± 0.161	0.315
	Maturation	UC	2.66 ± 0.134 b	3.16 ± 0.048 a	2.81 ± 0.165 b	2.82 ± 0.079 b	< 0.001
		EU	15.8 ± 1.74 a	12.4 ± 1.21 b	13.4 ± 1.08 b	12.3 ± 1.36 b	0.001
		LE	13.1 ± 1.41	10.6 ± 2.14	12.0 ± 1.47	12.6 ± 1.82	0.011
		LC	2.14 ± 0.322	2.56 ± 0.316	2.19 ± 0.127	2.26 ± 0.219	0.049
		TL	127.5 ± 10.8	122.9 ± 9.43	131.1 ± 7.03	128.8 ± 11.7	0.542
		PP/SP	0.936 ± 0.157	0.990 ± 0.160	1.02 ± 0.193	0.809 ± 0.088	0.114

			Control	MiKS 2	MiKS 3	MiKS 4	
*2024*	Veraison	UC	3.60 ± 0.447b	4.37 ± 0.607 a	4.71 ± 0.597 a	4.49 ± 0.547 a	< 0.001
		UE	15.4 ± 2.96 a	12.0 ± 2.60 b	12.47 ± 2.78 ab	14.53 ± 3.70 ab	0.023
		LE	14.4 ± 3.83	13.7 ± 2.56	15.3 ± 3.35	13.5 ± 2.25	0.48
		LC	3.16 ± 0.456 b	3.86 ± 0.557 a	3.76 ± 0.444 a	3.75 ± 0.668 a	0.01
		TL	175.9 ± 11.5	188.9 ± 22.5	179.3 ± 15.3	169.8 ± 12.3	0.042
		PP/SP	0.789 ± 0.257	0.540 ± 0.096	0.580 ± 0.060	0.648 ± 0.185	0.341
	Maturation	UC	3.10 ± 0.334 b	4.33 ± 0.588 a	4.62 ± 0.504 a	4.64 ± 0.398 a	< 0.001
		UE	15.5 ± 3.52 a	13.4 ± 2.62 ab	11.4 ± 2.45 b	12.6 ± 2.14 ab	0.005
		LE	14.9 ± 4.40	12.4 ± 2.46	14.3 ± 3.92	13.4 ± 2.16	0.304
		LC	2.63 ± 0.421 b	3.63 ± 0.446 a	3.93 ± 0.629 a	3.52 ± 0.383 a	< 0.001
		TL	164.0 ± 13.9	178.6 ± 18.4	181.7 ± 22.3	178.5 ± 15.3	0.083
		PP/SP	0.641 ± 0.103	0.658 ± 0.178	0.840 ± 0.196	0.647 ± 0.178	0.234

In 2024, both upper and lower cuticle thickness were further enhanced. At veraison, upper cuticle thickness increased by 21.4 to 30.8% in MiKS 2, MiKS 3 and MiKS 4, while at harvest, values reached up to 45% in same treatments. Similar response was observed for lower cuticle thickness, with increases of up to 49.4% in MiKS 3. In contrast, control vines exhibited a thicker upper epidermis (up to 22% in 2023 and 26% in 2024), compared to the MiKS-treated vines. No significant differences were observed in the leaf palisade/spongy ratio among MiKS treatments.

As shown in Table 2, the foliar MiKS applications significantly influenced leaf photosynthetic pigments and several biochemical parameters in both seasons.

**Table 2:** Effects of foliar application of different kaolin (Kl) and silicon (Si) formulations (MiKS): control (CTRL, water application), MiKS 1 (Kl 2%+Si 2%); MiKS 2 (Kl 2%+Si 4%); MiKS 3 (Kl 2%+Si 6%) and MiKS 4 (Kl 2%+Si 8%) on the photosynthetic pigments; chlorophyll a (Chl a, mg g⁻¹ DW), total chlorophylls (Chl_(a+b),_ mg g⁻¹ DW), chlorophyll a/b ratio (Chl_a/b_), total carotenoids (Car, mg g⁻¹ DW) and leaf metabolites concentration (Protein (mg g⁻¹ DW), Total soluble sugars (TSS, mg g⁻¹ DW), Proline (mg g⁻¹ DW), Total phenolic compounds (TPC, mg g⁻¹ DW); Hydrogen peroxide (H_2_O_2_; µmol g^− 1^) and Reactive oxygen species (ROS, nM DCF mg^− 1^ protein) in leaves “Touriga Nacional” grapevine variety at two phenological stages (veraison and harvest) during two growing seasons: 2023 and 2024. Values are expressed as mean ± standard deviation of six replicates (*n* = 6). Different letters indicate significant differences among treatments within the same phenological stage (Tukey’s test, *p* < 0.05). The absence of letters indicates no significant differences

Season	Phenological	Parameter	Foliar treatment					*p*-value
	Stage		Control	MiKS 1	MiKS 2		MiKS 3	
*2023*	Veraison	Chl a	1.81 ± 0.069 b	2.10 ± 0.063 a	2.15 ± 0.109 a	1.51 ± 0.038 c		< 0.001
		Chl_(a+b)_	3.53 ± 0.130 a	3.85 ± 0.235 a	3.94 ± 0.145 a	2.96 ± 0.085 b		< 0.001
		Chl_a/b_	1.06 ± 0.018 b	1.17 ± 0.066 a	1.20 ± 0.038 a	1.04 ± 0.023 b		0.002
		Car	0.560 ± 0.047 a	0.592 ± 0.009 a	0.613 ± 0.056 a	0.369 ± 0.026 b		< 0.001
		Protein	5.06 ± 0.423	5.25 ± 0.346	4.67 ± 0.099	4.89 ± 0.350		0.252
		TSS	6.45 ± 0.243	6.47 ± 0.202	5.86 ± 0.865	7.41 ± 1.157		0.192
		Proline	5.09 ± 0.644	5.30 ± 0.423	5.21 ± 0.238	5.13 ± 0.195		0.871
		TPC	20.5 ± 2.06ab	21.9 ± 0.541 a	18.5 ± 0.454 b	18.9 ± 0.713b		0.004
		H_2_O_2_	232.4 ± 50.6	215.4 ± 17.1	192.3 ± 47.5	220.7 ± 29.1		0.529
		ROS	0.090 ± 0.018 a	0.082 ± 0.010ab	0.057 ± 0.017bc	0.052 ± 0.007 c		0.005
	Harvest	Chl a	1.27 ± 0.023 c	1.45 ± 0.050 a	1.46 ± 0.060 a	1.13 ± 0.051 b		< 0.001
		Chl_(a+b)_	2.70 ± 0.023 b	2.95 ± 0.076 a	2.88 ± 0.081 ab	2.45 ± 0.090 c		< 0.001
		Chl_a/b_	0.897 ± 0.040 bc	0.972 ± 0.021 ab	1.02 ± 0.035 a	0.863 ± 0.015 c		< 0.001
		Car	0.360 ± 0.006 a	0.343 ± 0.034 a	0.362 ± 0.029 a	0.170 ± 0.017 c		< 0.001
		Protein	5.13 ± 0.436	4.58 ± 0.096	5.10 ± 0.229	4.94 ± 0.146		0.106
		TSS	6.28 ± 0.297	5.99 ± 0.471	6.74 ± 0.495	6.65 ± 0.405		0.161
		Proline	5.46 ± 0.154 a	4.26 ± 0.453 b	4.77 ± 0.160 b	4.57 ± 0.235 b		< 0.001
		TPC	21.8 ± 2.80	22.0 ± 0.863	19.9 ± 1.69	19.1 ± 0.902		0.092
		H_2_O_2_	283.3 ± 45.1 a	150.4 ± 19.6b	254.3 ± 62.2a	237.6 ± 41.6ab		0.001
		ROS	0.064 ± 0.009	0.062 ± 0.004	0.055 ± 0.003	0.048 ± 0.016		0.108

		**Parameter**	**Control**	**MiKS 2**	**MiKS 3**	**MiKS 4**		***p-value***
*2024*	Veraison	Chl a	1.36 ± 0.318 b	1.56 ± 0.094ab	1.77 ± 0.037 a	1.72 ± 0.094 ab		0.025
		Chl_(a+b)_	2.82 ± 0.257 b	3.23 ± 0.225ab	3.45 ± 0.080 a	3.46 ± 0.184 a		0.002
		Chl_a/b_	0.795 ± 0.189 b	0.937 ± 0.031 ab	1.00 ± 0.056 a	0.985 ± 0.029 ab		0.046
		Car	0.381 ± 0.120	0.394 ± 0.023	0.501 ± 0.033	0.459 ± 0.004		0.064
		Protein	5.25 ± 0.388	5.48 ± 0.344	5.91 ± 0.252	5.30 ± 0.232		0.112
		TSS	6.56 ± 0.976 ab	8.80 ± 0.649 a	6.10 ± 1.398 b	6.71 ± 0.107 ab		0.028
		Proline	5.32 ± 0.640 a	4.33 ± 0.411 b	4.26 ± 0.275 b	4.08 ± 0.275 b		0.001
		TPC	19.7 ± 1.37	21.6 ± 2.71	20.9 ± 1.80	21.9 ± 1.14		0.364
		H_2_O_2_	337.9 ± 55.7 a	193.9 ± 37.9 b	181.0 ± 15.4 b	262.0 ± 13.7ab		0.001
		ROS	0.131 ± 0.027	0.110 ± 0.012	0.096 ± 0.011	0.121 ± 0.009		0.053
	Harvest	Chl a	1.16 ± 0.023	1.45 ± 0.210	1.35 ± 0.222	1.47 ± 0.127		0.070
		Chl(_a+b_)	2.66 ± 0.015 c	3.33 ± 0.278 ab	2.78 ± 0.071 bc	3.69 ± 0.497 a		< 0.001
		Chl_a/b_	0.760 ± 0.006 a	0.650 ± 0.041 b	0.777 ± 0.040 a	0.726 ± 0.024 a		< 0.001
		Car	0.139 ± 0.015 b	0.109 ± 0.021 b	0.215 ± 0.016 a	0.140 ± 0.007 b		< 0.001
		Protein	4.37 ± 0.367 b	4.07 ± 0.455 b	4.36 ± 0.808 b	5.71 ± 0.260 a		0.018
		TSS	8.51 ± 0.979	7.85 ± 0.695	7.26 ± 0.538	7.08 ± 0.425		0.124
		Proline	5.79 ± 0.328 a	4.45 ± 0.620 b	5.11 ± 0.186 ab	5.35 ± 0.131 a		0.002
		TPC	22.8 ± 2.48	20.3 ± 3.28	20.8 ± 1.47	20.5 ± 1.56		0.429
		H_2_O_2_	344.4 ± 20.2	339.4 ± 35.3	276.2 ± 15.3	295.3 ± 60.9		0.100
		ROS	0.162 ± 0.031	0.132 ± 0.059	0.103 ± 0.026	0.123 ± 0.021		0.217

In 2023, at veraison, MiKS 1 and MiKS 2 promoted an overall enhancement of Chl *a* (16.1 and 18.8%), Chl _(a+b)_ (9.1 and 11.6%), and Chl *a/b* ratio (10.4% and 13.7%, respectively) compared to control vines. In contrast, MiKS 3 showed the opposite effects, with decreases of 16% in Chl *a* and Chl _(a+b),_ and 34.1% in carotenoids. These patterns persisted at harvest, with MiKS 1 and MiKS 2 maintaining higher Chl *a* content (up to 15%), while MiKS 3 continued to exhibit lower pigment content. Only MiKS 1 showed a significant increase in Chl _(a+b)_ (9.3%), while carotenoid content remained lowest in MiKS 3, decreasing 52%.

In 2024, pigments content was again significantly influenced by MiKS treatments. MiKS 3 and MiKS 4 consistently enhanced leaf pigments across both phenological stages, with increases of around 30% in Chl *a*, 23% in total Chl _(a+b),_ and 26% in the Chl *a/b* ratio at veraison, and maintaining higher contents at harvest, where Chl *a* by 16–27% and total Chl _(a+b)_ by up to 39% compared with the control.

Regarding carotenoids, MiKS 3 consistently maintained the highest content across veraison and harvest, demonstrating increases of 32% and 54.7%, respectively. Interestingly, MiKS 2 showed a reduction in the Chl a/b ratio (14.5%), while MiKS 3 exhibited the highest carotenoid content, showing a 54.7% increase compared with the control.

Total protein content was not significantly influenced in 2023 or at veraison in 2024. A significant effect was observed only at harvest in 2024, when MiKS4 treated vine showed higher protein content (31%) compared with the control. Total soluble sugars (TSS) were not significantly affected by the MiKS treatments in 2023 or at harvest in 2024. However, in the veraison in 2024, TSS differed significantly among treatments, with MiKS 2 showing values 44.3% higher than MiKS 3. Proline content showed no significant differences in the veraison in 2023, but at harvest in the same year it was significantly reduced in all MiKS treatments, by 22.0%, 12.6%, and 16.3% in MiKS 1, MiKS 2, and MiKS 3, respectively. In 2024, proline was significantly reduced in all MiKS treatments at veraison, with decreases of 18.6%, 19.9%, and 23.3% in MiKS 2, MiKS 3, and MiKS 4, respectively. At harvest, a significant reduction was observed only in MiKS 2 (23.1%).

Regarding antioxidant response, total phenolic compounds (TPC) were significantly only at veraison in 2023, with decreases of 9.8% and 7.8% in MiKS 2 and MiKS 3, respectively, compared to control. In turn, H₂O₂ content was significantly reduced at harvest in 2023 only in MiKS 1, by 46.9% compared to control. In 2024, at veraison, H₂O₂ was also significantly lower in MiKS 2 and MiKS 3, with reductions of 42.6% and 46.4%, respectively, while no significant differences were observed at harvest. Similarly, ROS content was significantly reduced only at veraison in 2023, with reductions of 36.7% and 42.2% in MiKS 2 and MiKS 3, while no significant differences were observed at harvest in 2023 or at both phenological stages in 2024.

### Leaf nutrient balance and yield component effects under kaolin and silicon mixtures application

Leaf mineral composition, assessed in 2023, was largely unaffected by MiKS formulations, as no significant differences were observed for macronutrients (N, P, K, Ca and Mg) or for micronutrients (B, Fe and Zn) (Table 3). In contrast, sulfur (S), manganese (Mn), and copper (Cu) concentrations were significantly influenced by foliar treatments. While MiKS1 and MiKS2 showed values comparable to the control, vines treated with MiKS3 exhibited significantly lower Mn, Cu and S concentrations, corresponding to reductions of approximately 45.7%, 52.2% and 19.6%, respectively, compared with the control.

**Table 3:** Effects of foliar application of different kaolin (Kl) and silicon (Si) formulations (MiKS): control (CTRL, water application), MiKS 1 (Kl 2%+Si 2%); MiKS 2 (Kl 2%+Si 4%); MiKS 3 (Kl 2%+Si 6%) and on macronutrients (N, P, K, Ca, Mg; mg g^− 1^) and micronutrients (B, Cu, Fe, Zn, Mn; mg g^− 1^ DW) the concentration in petioles of “Touriga Nacional” variety leaves, in 2023 growing season. Values are expressed as mean ± standard deviation of three replicates (*n* = 3), each replicate corresponding to one composite sample of five petioles per block. Different letters indicate significant differences among treatments (*p* < 0.05). The absence of letters indicates no significant differences

Mineral	Foliar Treatment				*p*-value
	Control	MiKS 1	MiKS 2	MiKS 3	
macronutrients					
N	4.65 ± 0.746	4.44 ± 0.709	4.37 ± 0.812	4.62 ± 1.01	0.969
P	2.03 ± 0.407	2.00 ± 0.667	1.76 ± 0.335	1.63 ± 0.335	0.677
K	3.32 ± 0.946	3.46 ± 0.721	3.08 ± 1.18	4.70 ± 1.36	0.325
Ca	20.4 ± 0.934	20.3 ± 1.99	20.2 ± 1.89	18.8 ± 2.60	0.723
Mg	19.3 ± 1.63	17.5 ± 2.18	19.9 ± 2.85	16.1 ± 0.594	0.149
S	0.908 ± 0.031 ab	0.929 ± 0.110 a	0.879 ± 0.082 ab	0.730 ± 0.049 b	0.043
micronutrients					
B	18.5 ± 1.52	17.9 ± 2.09	18.8 ± 2.42	15.8 ± 0.070	0.240
Fe	337.6 ± 160.5	563.7 ± 52.5	361.0 ± 86.1	443.7 ± 160.8	0.188
Zn	88.9 ± 15.7	85.2 ± 3.84	160.2 ± 42.4	175.9 ± 85.3	0.103
Mn	963.9 ± 63.2 a	935.2 ± 28.0 a	1072.7 ± 177.1 a	523.6 ± 131.1 b	0.001
Cu	58.8 ± 8.09 a	43.8 ± 10.17 ab	40.4 ± 5.26 ab	28.1 ± 1.66 b	0.005

**Fig. 6:**
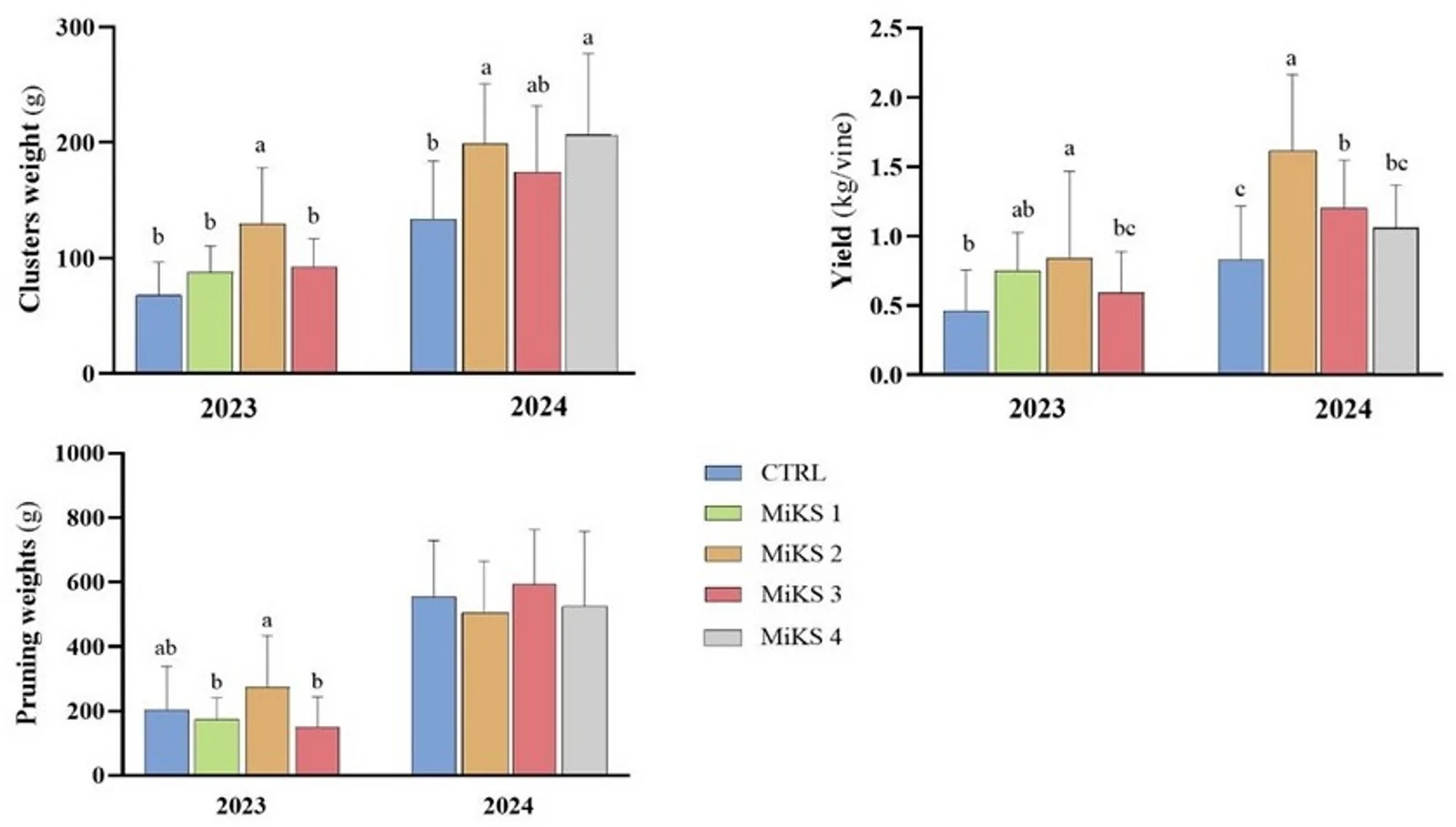
Effects of foliar application of different kaolin (Kl) and silicon (Si) formulations: control (CTRL, water application), MiKS 1 (Kl 2%+Si 2%); MiKS 2 (Kl 2%+Si 4%); MiKS 3 (Kl 2%+Si 6%) and MiKS 4 (Kl 2%+Si 8%) on yield performance and vine balance over two growing seasons (2023 and 2024). (**a**) cluster weight (g), (**b**) yield (kg per vine), and (**c**) pruning weight (g). Bars represent mean ± standard deviation of fifteen replicates (*n* = 15). Different letters indicate significant differences among treatments within the same growing season (Tukey’s test, *p* < 0.05). The absence of letters indicates no significant differences

In both growing seasons, MiKS treatments influenced yield (kg vine⁻¹) and bunch weight (g) (Fig. 6a, b). In 2023, MiKS 2 showed the highest yield performance, increasing bunch weight from 67.5 to 129.7 g (91.1%) and yield from 0.46 to 0.84 kg per vine (82.6%), compared with control. This trend continued in 2024, with MiKS 2 increase yield from 0.83 to 1.61 kg vine⁻¹ (94.6%), followed by MiKS 3, in which the increase was from 0.83 to 1.20 kg vine⁻¹ (44.3%). Similarly, bunch weight increased under MiKS 2 and MiKS 4, with this last showing the highest values, reaching 199.1 g (48.7%) and 206.9 g (54.5%), respectively, compared with 133.9 g in the control.

Regarding pruning wood weight (Fig. 6c), significant differences among treatments were observed in 2023. MiKS 2 induced the highest biomass accumulation, corresponding to an increase of 35% relative to control vines and 82% relative to the MiKS 3, which showed the lowest values. In contrast, no significant differences were observed among treatments in 2024, although pruning wood weight was generally higher than in the previous season.

## Discussion

### Leaf physiological outputs

Foliar application of Kl and Si have been widely recognized for their capacity to mitigate (a)biotic stress in plants, contributing to the maintenance of physiological and metabolic balance [9–11, 23, 45, 46]. Although individual benefits of Kl and Si are well documented, and their combined use has been previously explored [47], their combined effects on grapevine performance under Mediterranean conditions, particularly in Touriga Nacional, remains insufficiently understood.

MiKS treatments were evaluated over two growing seasons characterized by distinct climatic conditions; 2023 was comparatively milder, whereas 2024 experienced a higher frequency of days with extreme temperatures (> 35 °C). These climatic conditions allowed the assessment of MiKS performance under increasingly harsh summer in the Douro Demarcated Region, which are expected to intensify under ongoing climate change.

The predawn leaf water potential (Ψ_pd_), is a key indicator of grapevine water status and plant response to deficit water [48–50]. As summer progressed, increasing air temperature and decreasing soil water availability led to more negative Ψ_pd_ in control grapevines, indicating moderate to severe water stress. In contrast, MiKS treated vines maintained significantly higher Ψ_pd_ across both seasons, suggesting an improved plant water status.

Among the treatments, MiKS 3 tended to maintain higher Ψ_pd_ even under elevated air temperature and high vapor pressure deficit (VPD) conditions, suggesting a better maintenance of plant water status under combined atmospheric and soil water limitations [49].

The slightly higher Ψ_pd_ observed in 2024, despite warmer conditions and lower cumulative rainfall during the same period, may be related to soil water availability and atmospheric demand.

In fact, winter was characterized by high rainfall, resulting in adequate soil water availability that remained close to field capacity until April, ensuring optimal water conditions at the onset of the growing season. In addition, moderate VPD during veraison (4.1 kPa) and harvest (4.0 kPa) allowed grapevines to regulate transpiration through partial stomatal closure, thereby limiting water loss and allowed overnight tissue rehydration [51]. This response may contribute to the higher Ψ_pd_ observed across all MiKS treatments.

The better plant water status promoted observed in MiKS treatments was accompanied by improved in grapevine performance, particularly in terms of gas exchange and PSII photochemical efficiency [52]. Under drought conditions, stomatal closure is a common adaptive response to limit transpirational water loss. However, it also restricts CO₂ uptake into the leaf, thereby constraining photosynthesis [53, 54]. In addition, reduced transpiration can increase leaf temperatures, potentially exacerbating oxidative stress and affecting multiple physiological processes, including enzyme activity, stomatal regulation, and water-use efficiency [55, 56].

These findings suggest that foliar MiKS applications were associated with the maintenance of gas exchange, particularly during veraison, a phenological stage highly sensitive to abiotic stress.

Among the treatments, MiKS 2 and MiKS 3 were most effective in increasing *A* and *g*_*s*_ without compromising the *A/g*_*s*_, indicating a balanced physiological response under summer stress conditions in the Douro region. Although increased *g*_*s*_ is generally associated with higher transpirational water loss, the simultaneous increase in *A* observed in MiKS- treated vines, resulted in maintained or improved *A/g*_*s*_, suggesting an efficient balance between carbon assimilation and water loss [9, 57]. These effects were more pronounced during morning measurements, particularly for *A* and *A/g*_*s*_, suggesting a more efficient physiological response, with enhanced carbon assimilation before stomatal closure under elevated leaf temperatures and VPD [58, 59].

These responses are in line with previously reported effects of Kl and Si. Kaolin acts as a radiation reflective barrier that reduce incident radiation and leaf temperature, while silicon has been associated with improved gas exchange and stress tolerance in grapevines [17, 60–63]. When combined, Kl and Si may have contributed to the maintenance of carbon assimilation and stomatal function, regulating leaf temperature and improved physiological performance under Mediterranean summer stress conditions [17, 60, 61].

Several studies have reported that Si application enhances gas exchange (A, gs, and A/gs) under stress conditions, although responses may vary depending on grapevine genotype and environmental conditions [55, 63–65].

Consistent with these responses, chlorophyll *a* fluorescence parameters indicated that MiKS treatments were able to maintain photosystem II (PSII) functionality under summer stress. MiKS 2 and MiKS 3 treated vines showed higher effective *Φ*_*PSII*_, greater *qP*, and lower NPQ particularly at midday during veraison (2023), indicating a more efficient light utilization for photochemistry rather than thermal dissipation [64].

The *F*_*v*_/*F*_*m*_ remained close to the optimal values (0.83) across treatments, particularly during the morning at the veraison (2023) and at midday during veraison (2024), suggesting no clear evidence of photoinhibition [64]. Overall, the combination of higher *qP*, and ΦPSII together with lower NPQ, indicates sustained photochemical activity, lower degree of photo-oxidative stress, and improved preservation of leaf tissue integrity under summer stress conditions [65, 66].

### Morphological and biochemical responses

Beyond the physiological responses, MiKS treatments induced structural and biochemical changes in grapevine leaves.

Morphological analysis showed that MiKS treatments promoted structural modifications, most prominently in cuticle thickness. The significant increase in upper cuticle thickness, particularly under MiKS 2 and MiKS 3, may contribute to the reinforcement of the leaf surface. This effect is consistent with previous report describing Si deposition beneath the cuticle and within cell walls, in other plant species, where it contributes to the formation of silica-cellulose and silica-cuticle bilayers that reinforce the leaf surface [67–69]. 

As result, the thicker cuticle may act as an additional physical barrier, and could be associated with reduced non-stomatal water loss and enhanced plant water retention, which is particularly relevant under severe water deficit conditions, such as those frequently observed in the Douro region [70, 71].

In addition, these structural modifications may enhance leaf mechanical strength and improve protection against pathogen entry, as thicker layers can limit pathogen penetration [27, 72–74], . These observations are consistent with previous studies describing the role of Si in reinforcing plant tissues under drought conditions [9, 67, 75, 76]. For instance, in *Festuca arundinacea*, Si nanoparticles have been show to increase cuticle thickness thought Si deposition of beneath the cuticle and within the cell walls [68].Previous studies have shown that kaolin application alone can induce morphological changes, often associated with reduced leaf thickness due to its reflective properties. In olive trees, Kl-treated plants developed thinner leaves, characterized by reduced cuticle and epidermal thickness, attributed to the shading effect of the Kl film on leaf surface, which increases light reflectance and reduces light absorption [14, 22, 23]. When combined with Si, this effect may be counterbalance though the potential reinforcing cellular structures [77].

Leaf metabolites further support the protective role of MiKS treatments. Photosynthetic pigments are key indicators of photosynthetic performance and photoprotection [78–81]. The increases in Chl *a*, Chl _(a + b),_ Chl *a/b* ratio, and carotenoid content observed in MiKS-treated vines suggest enhanced stability of the photosynthetic apparatus under high temperature and radiation conditions, potentially reducing pigment degradation.

This is particularly relevant, as high temperatures (> 35 °C) are known to accelerate chlorophyll degradation and impair RuBisCO activity through the thermal sensitivity of RuBisCO activase, ultimately reducing carbon assimilation and photosynthetic efficiency [82–84]. The increase in the Chl *a/b* ratio may indicate adjustments in light-use efficiency system, potentially involving a reduction in light-harvesting antennae size and improved energy distribution within PSII, which are typically associated with acclimation to high irradiance [37, 85]. Furthermore, carotenoids responses support the idea of enhanced photoprotection, particularly in MiKS 3 and MiKS 4. These pigments contribute to the dissipation of excess energy and scavenging ROS, thereby protecting photosynthetic structures under stress conditions [86, 87].

The reduction in oxidative stress markers, such as hydrogen peroxide (H₂O₂) and ROS, further supports the hypothesis that MiKS treatments, specially MiKS 2 and MiKS 3, may be associated with an improved redox balance. Conversely, the higher ROS content observed in control vines, especially during veraison, indicates that, in the absence of foliar protectors, these vines are subjected to greater oxidative stress. Under stress conditions, when light absorption exceeds the capacity of the photosynthetic machinery, ROS production may overwhelm the antioxidant defence system, leading to cellular damage [78, 88, 89].

Proline accumulation is a common response to osmotic and oxidative stress, acting as an osmolyte, antioxidant and signalling molecule involved in membrane and protein stabilization [90]. The lower proline content observed in MiKS treated vines, particularly in MiKS 2 and MiKS 3 compared to control vines, may indicate a reduced stress perception or an improved stress tolerance, suggests that MiKS treatments attenuated the need for osmotic adjustment mechanisms summer stress conditions [43, 90–92].

Similarly, TPC showed a significant reduction in specific MiKS treatments at veraison in 2023, while no differences were observed at the other phenological stages and seasons, suggesting a reduced involvement of phenolic compounds in the antioxidant response.

These responses are consistent with the complementary roles of Kl and Si. Kaolin reduces excess radiation and heat load, thereby limiting ROS formation, while Si contributes to antioxidant defence by stabilizing cellular membranes and enhancing enzymatic detoxification systems. Similar effects of Si in mitigating oxidative stress have been reported in grapevine and other crops [93, 94].

### Leaf minerals and production

Leaf nutrient profile reflects both soil nutrient availability and vine uptake efficiency, providing an indicator of grapevine nutritional status [95]. MiKS treatments did not significantly alter most macro and micronutrients content in the leaf profile assessed in 2023.

However, vines treated with MiKS 3 showed a significant reduction in Mn, S and Cu content.

This decreased may have associated with the reduced chlorophyll *a*, and carotenoid contents observed under MiKS3 in 2023. Manganese serve as an essential cofactor in the water-oxidizing Mn₄CaO₅ cluster of photosystem II, while copper is required for plastocyanin-mediated electron transport between the cytochrome b₆f complex and photosystem I [96–98]. In addition, sulfur plays a critical role in the structure of iron–sulfur (Fe–S) cluster proteins, such as ferredoxin, which are essential for electron transport in photosystem I [99]. Together, these elements are key cofactors in enzymatic processes related to chlorophyll biosynthesis and maintenance of photosynthetic function [100, 101].

This reduction in micronutrient availability may be related to possible silicon-mediated redistribution processes within leaf tissues. Silicon can influence nutrient mobility and partitioning, particularly of divalent cations such as Mn²⁺ and Cu²⁺, either through interactions in the apoplast or by affecting their translocation via the phloem [102–104]. As a result, these processes may selectively limit the availability of certain micronutrients, including Mn, Cu and S, in actively developing tissues. Similar responses have been reported in species such as tomato [105], orange [106], and cucumber leaves [103].

Despite reduced pigment content, MiKS 3 vines did not exhibit significant increase in ROS or H_2_O_2_ levels, suggesting that pigment reduction may be related to metabolic adjustments rather than oxidative degradation.

The increase in yield observed under MiKS 2 may be associated with changes in individual bunch weight. In 2023, this response was accompanied by higher pruning wood biomass in 2023. In contrast, in 2024, yield improvements occurred without significant changes in pruning weight, which may indicate a more efficient allocation of assimilates towards reproductive organs. This pattern suggests a possible adjustment in source–sink relationships, potentially supported by improved physiological performance under MiKS treatments.

## Conclusion

The results of the present study demonstrate that foliar formulations combining Kl and Si (MiKS) were associated with favourable physiological, biochemical and morphological responses in “Touriga Nacional” under summer stress conditions. MiKS 2 and MiKS 3, was frequently associated with higher plant water status, photosynthetic performance, increased cuticle thickness and better preservation of photosynthetic pigments in comparison with control. Overall, the results suggest that Kl-Si formulations, particularly those containing 4–6% Si, may help support grapevine physiological performance during periods of elevated summer stress.

Conversely, increasing Si concentration to 8% (MiKS 4) did not result in consistent benefits compared to 6% Si (MiKS 3), suggesting that higher Si did not further improve treatments performance under the tested conditions. 

These findings highlight the potential relevance of MiKS treatments as a part of sustainable viticultural management strategies for Mediterranean environments. However, when integrated with other management practices, MiKS formulations may contribute to mitigating the effects of summer stress, being associated with maintained plant physiological performance, without negative effects on yield.

## Data Availability

All data generated or analyzed during this study are included in this manuscript.
